# Time to Recovery from COVID-19 and Its Predictors in Patients Hospitalized at Tibebe Ghion Specialized Hospital Care and Treatment Center, A Retrospective Follow-Up Study, North West Ethiopia

**DOI:** 10.1155/2023/5586353

**Published:** 2023-09-12

**Authors:** Desiyalew Habtamu Tamiru, Abebaw Gedef Azene, Gebeyaw Wudie Tsegaye, Kebadnew Mulatu Mihretie, Samuel Hunegnaw Asmare, Wudneh Arega Gete, Simachew Animen Bante

**Affiliations:** ^1^Department of Public Health Emergency, Humedica e.V International Aid Organization, Addis Ababa, Ethiopia; ^2^Department of Epidemiology and Biostatistics, College of Medicine and Health Sciences, Bahir Dar University, Bahir Dar, Ethiopia; ^3^Department of Internal Medicine, College of Medicine and Health Sciences, Bahir Dar University, Bahir Dar, Ethiopia; ^4^Department of Communicable Disease Control (CDC), Bahir Dar, Ethiopia; ^5^Department of Midwifery, College of Medicine and Health Sciences, Bahir Dar University, Bahir Dar, Ethiopia

## Abstract

**Background:**

Since the end of 2019, the world has been facing a new coronavirus disease 19 (COVID-19), which is considered a global pandemic. COVID-19 is considered a major public health burden due to the uncontrolled morbidity and mortality of the global community. The World Health Organization estimates the recovery time as 2 weeks for patients with mild infection and 3 to 6 weeks for those with serious illnesses. The recovery time and its predictors are not well studied in Ethiopia yet. Therefore, the aim of this study was to estimate time to recovery from COVID-19 and its predictors among COVID-19 patients admitted to Tibebe Ghion Specialized Hospital care and treatment center, North West Ethiopia.

**Methods:**

An institution-based retrospective follow-up study was conducted among 452 COVID-19 patients admitted to Tibebe Ghion Specialized Hospital from March 2020 to September 2021. Simple random sampling using a table of random number generators was used to select study units. Data entry and analysis were performed using EpiData 3.1 and Stata version 14, respectively. Bivariable and multivariable Cox proportional hazard analyses were used to identify predictors of recovery time. An AHR at a 5% level of significance was used to identify significant predictors.

**Results:**

: Among 452 COVID-19 patients, 437 (88%) were recovered, with a median recovery time of 9 days. Recovery time was significantly related to age (AHR = 0.98; 95% CI = 0.97, 0.99), oxygen saturation (AHR = 0.42; 95% CI = 0.31, 0.56), shortness of breath (AHR = 0.65; 95% CI = 0.47, 0.85), disease severity (moderate (AHR = 0.63; 95% CI = 0.47, 0.85) and severe (AHR = 0.32; 95% CI = 0.22, 0.47)), and comorbidities (AHR = 0.67; 95% CI = 0.53, 0.84). *Conclusions and recommendations*: The overall median recovery time was 9 days. Older age, low oxygen saturation, shortness of breath, disease severity (moderate and severe), history of comorbidities, and high-level of WBC were predictors of delayed recovery time. On the other hand, corticosteroid use significantly shortens the median recovery time of COVID-19 patients. Thus, patients presented with older age, low oxygen saturation, shortness of breath, moderate and severe COVID-19 disease, comorbidities, and increased WBC need to be closely monitoring and followed up by healthcare providers. In addition, there should be special attention during the administration of corticosteroid.

## 1. Introduction

Since the end of 2019, the world has been facing a new coronavirus disease (COVID-19), which is considered a global pandemic. The disease is caused by the severe acute respiratory syndrome coronavirus 2 (SARS-CoV-2), which is a highly contagious and fatal strain of coronavirus disease [1]. The majority of the early cases in Wuhan City, China, were found to have been exposed to the wet animal market, suggesting a zoonotic origin [1, 2]. COVID-19 is a substantial public health concern and it could be considered the most complex global challenge [2, 3].

COVID-19 spreads faster than its two ancestors, Middle East Respiratory Syndrome Coronavirus (MERS-CoV) and Severe Acute Respiratory Syndrome Coronavirus (SARS-CoV), but with a lower fatality rate [4, 5]. COVID-19's spread has been devastating for the millions of individuals who have been infected and died as well as for many others who have lost their jobs [6].

Reports indicated that COVID-19 has a health, economic, social, and psychological impact despite its clear global impact yet uncertain as cases and deaths are likely to emerge [4, 7]. The health impact is the most devastating, affecting human health and health care, systems, directly changing patterns of morbidity and mortality across countries [6].

Extensive precautions have been implemented to reduce the transmission and control the current outbreak. All countries have taken different actions based on the World Health Organization's (WHO) general protocol or country-specific guidelines [1].

In Ethiopia, the situation of the disease changes from time to time as the spread of the disease is not under control yet. Since its date of occurrence, in March 2020, the government has been implementing different measures as part of a rapid response, like restricting any public gatherings, including school and sporting activities. Despite the fact that the disease is not under control, all preventive efforts are currently loosely implemented [8, 9].

Time to recovery is the time between dates of confirmed COVID-19 infection to recovery from the disease. The average median recovery time of COVID-19 varies among patients and settings [10]. According to WHO, individuals with moderate infections could expect to recover in two weeks, while those with serious illnesses can expect to recover in three to six weeks [11]. Based on evidences obtained from few studies in Ethiopia, the recovery time was longer than that of the WHO findings [12–14].

Previous studies conducted worldwide indicated that the median recovery time of COVID-19 patients was determined by age, sex, oxygen saturation at admission, dyspnea, COVID-19 severity, history of comorbidities, baseline creatinine, hemoglobin and WBC levels and use of drugs like corticosteroids and beta blockers [13–26].

Estimating disease recovery time and predictors is the best way for reasonable resource allocation and setup organization in the case of a novel disease with no known treatment. The increase in the number of inpatient admissions and delayed in the recovery time creates hospital overcrowding. As a result of healthcare staff spending much time fighting COVID-19, other medical services would receive less attention, exacerbating the existing inadequate health care system of poor countries like Ethiopia [12, 27]. The healthcare institution costs are dependent not only on the interventions administered but also on the time taken to recover [28, 29]. Longer recovery times require more healthcare providers and hospital beds, resulting in overcrowded hospitals and a shortage of life-saving oxygen and other medical supplies, which is especially true in developing countries like Ethiopia [30–32]. As a result, identifying predictors of recovery time is critical. This is due to the fact that healthcare workers could provide care to patients based on their unique clinical needs, allowing them to recover faster and lowering healthcare expenses [33].

Many studies have been undertaken around the world to better understand disease recovery time and predictors, but only a few have been conducted in Ethiopia. Our country's fundamental population characteristics, economic conditions, healthcare structure, and endemic illness patterns differ from those of other countries, making it difficult to generalize findings based on a few research or findings from other parts of the world.

Therefore, estimating the recovery time and its predictors, in the Ethiopian context, is very essential for proper distribution and utilization of human and material resources for better patient care. Hence, this study aimed to estimate the recovery time and its predictors among COVID-19 patients admitted at Tibebe Ghion Specialized Hospital (TGSH) COVID-19 care and treatment center.

## 2. Materials and Methods

### 2.1. Study Setting and Period

This study was conducted in TGSH COVID-19 care and treatment center, a teaching hospital under college of medicine and health sciences of Bahir Dar University located in Bahir Dar, Ethiopia. The study period was from March 2020 to September 2021, while the data extraction period was from October 1 to October 15, 2021.

#### 2.1.1. Study Design

A hospital-based retrospective follow-up study was conducted among COVID-19 patients admitted to TGSH care and treatment center.

#### 2.1.2. Sources and Study Population

The source population comprised all COVID-19 patients admitted to the TGSH care and treatment center with a confirmed diagnosis of COVID-19 using real-time reverse transcription polymerase chain reaction, whereas the study population comprised all COVID-19 patients admitted to the TGSH care and treatment center from March 2020 to September 2021.

#### 2.1.3. Inclusion and Exclusion Criteria

All COVID-19 patients who were admitted to TGSH care and treatment center during the study period were included, while COVID-19 patients' whose medical records had incomplete information on date of admission, date of event (recovery) occurred, and charts missed important predictor variables were excluded.

### 2.2. Study Variables

#### 2.2.1. Dependent Variables

Time to recover from COVID-19 (in days).

#### 2.2.2. Independent Variables

1. Sociodemographic factors: Age, sex, and residence.
2. Vital signs at admission: Blood pressure, respiratory rate, pulse rate, temperature, and oxygen saturation.
3. Presentation of symptoms: Fever, cough, myalgia/arthralgia, shortness of breath, headache, COVID-19 severity score, chest pain, and fatigue.
4. Presence of previous comorbidities: Diseases like chronic heart disease, hypertension (HTN), diabetes mellitus (DM), chronic kidney disease, asthma, tuberculosis, and HIV.
5. Baseline laboratory values: Hemoglobin (Hgb), white blood cell (WBC), red blood cell (RBC), platelet (PLT), and creatinine.
6. Treatment provided: Antibiotics, corticosteroids, unfractionated heparin, and oxygen supply.

### 2.3. Operational Definitions

  COVID-19 patient: Any patient who tested positive for COVID-19 antigen test as reported by a laboratory was given a mandate to test such patients by the Ethiopian Federal Ministry of Health [34].
  Asymptomatic patient: Any patient who tested positive for COVID-19 but did not have any symptoms [34].
  Recovery: Recovered from COVID-19 infection as evidenced by two negative RT-PCR tests done at least 24 hours apart.
  Event: Achieving recovery from COVID-19 infection.
  Censoring: Patients lost to follow-up, transferred out, died, and discharged against medical consent.
  Survival time: Time in days from the patient was diagnosed positive for COVID-19 by using RT-PCR test to the occurrence of the outcome, the event or censoring.
  Time to recovery: the time, in days, between the dates of laboratory confirmation of COVID-19 infection to recovery from COVID-19 infection.
  Lost: COVID-19 patients who left the hospital with unknown status of the outcome.
  Normal laboratory values: WBC (4000−10,000 cells/ml), RBC count (3.5–5.5) ∗10^6^ cells/ml), Hgb level (12 16 gm/dl female and 13–17 gm/dl male), platelet count ((150–450)∗10^3^ cells/ml), and creatinine level (0.6–1.2 mg/dl).
  Low oxygen saturation: Oxygen saturation less than 95%.

### 2.4. Sample Size Determination and Sampling Technique

The sample size was determined by using the sample size calculation formula for survival analysis by considering the following statistical assumptions: 95% confidence interval (CI), power of 90%, adjusted hazard ratio of 1.36 [13], and 5% marginal error. The final sample size was calculated to be 452, and a simple random sampling method using a table of random number generator was employed to select the study participants.

### 2.5. Data Collection Instrument

Data were extracted through document review using a structured checklist prepared in English. The data extraction tool was developed by adapting from the WHO case report form, by reviewing different literatures and after reviewing some selected patients' charts. The tool consists of sociodemographic predictors, vital signs at admission, sign and symptoms, presence of comorbidities, baseline laboratory investigations, and treatment given.

### 2.6. Data Management and Analysis Procedures

Data were entered using Epi-Data version 3.1 and analyzed using STATA version 15. Before analysis, data were cleaned and checked for consistency by using simple frequencies and cross tabulation; re-categorization of categorical variables and categorization of continuous variables was done to make it suitable for analysis. Descriptive statistics was used to present demographic and background clinical characteristics of the patients. The Kaplan–Meier survival curve was used to estimate median survival time and cumulative probability of survival, while log-rank test was used to assess overall survival differences between group predictors. Cox proportional hazard regression analysis was used to identify the potential predictors of recovery time. The proportional hazard assumption was assessed using both graphical and statistical methods [35, 36]. The graphical method was used to assess proportional hazard assumptions by plotting KM survivor probability curves versus survival time and analyzing whether the curves are approximately parallel or not. Schoenfeld's global goodness-of-fit test, on the other hand, was used to determine whether the hazard assumptions were met. An overall global goodness-of-fit test with a cut point of larger than 0.05 was used to declare the proportional hazard assumption was achieved [35, 37]. Multicollinearity between predictors was also checked. Bivariable Cox-proportional hazard regression model was fitted for each explanatory variable and those variables having *p*-value ≤0.25 were selected for multivariable Cox regression analysis. Adjusted hazard ratio (AHR) with 95% confidence intervals was computed and statistical significance was declared at *p* < 0.05. Finally, results were presented using tables, graphs, and text.

## 3. Results

### 3.1. Sociodemographic and Background Clinical Characteristics

The data were collected from the medical records of 452 COVID-19 patients. The patient's median age was 57.5 years, with an interquartile range of 30 years (37.5 to 67.5). 302 (66.8%) and 305 (67.5%) of the respondents were male and urban residents, respectively.

At admission, 318 (70.3%) patients presented with a normal respiratory rate. Similarly, 329 (72.8%), 403 (89.2%), and 374 (83.7%) were admitted with normal pulse rate, blood pressure, and temperature, respectively, whereas 236 (52.0%) patients were presented with low oxygen saturation. Four hundred twenty-two (93.4%) patients were presented with one or more COVID-19 related symptoms. One hundred fifty-three (33.8%) of the patients had one or more comorbidities. The most common comorbidities were diabetes mellitus, 72 (15.9%), hypertension, 63 (13.7%), and asthma, 21 (4.6%) **(**Table 1).

### 3.2. Baseline Laboratory Investigations

The main laboratory investigations that the admitted patients underwent were CBC (WBC, RBC, Hgb, and PLT count) and creatinine level. Three hundred one (66.6%) patients had a normal baseline WBC level while the rest, 37 (8.2%) and 114 (25.2%), had low and high levels of WBC. Similarly, 206 (45.6%), 46 (10.2%), and 200 (44.2%) of patients were investigated to have normal, low, and high levels of creatinine. The details are demonstrated in Table 1 (Table 1).

### 3.3. Types of Medical Treatment Given

The hospitalized COVID-19 patients received various treatments based on their clinical needs. Antibiotics were provided to 338 (74.8%) patients, while corticosteroids, UFH, and oxygen supplementation were given to 271 (60%), 66 (14.6%), and 245 (54%) patients, respectively.

### 3.4. Median Recovery Time and Survival Comparison between Group Predictors

The overall median recovery time was 9 days, with an interquartile range of 7 days and a 95% CI of 8–10 days. The majority of patients, 397 (87.8%), were recovered, while the remaining 55 (12.2%) were censored, with 37 (8.2%) dead and 18 (4%) being transferred out, discharged against medical consent, or lost.

**Figure 1:**
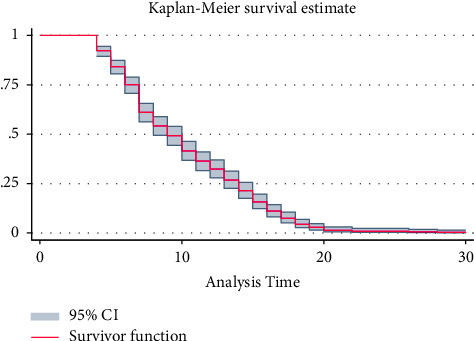
Overall Kaplan–Meier survival probability curve among COVID-19 patients, admitted to TGSH, North West Ethiopia, 2022 (*n* = 452).

For categorical predictors, survival differences were assessed using both the KM graphical curve and the log-rank test. The overall Kaplan–Meier survival curve indicated the fastest recovery was achieved in the earliest days of admission (Figure 1).

**Figure 2:**
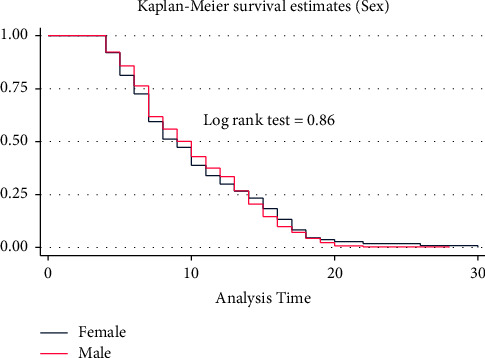
KM survival curve for sex among COVID-19 patients admitted to TGSH, North West Ethiopia, 2022 (*n* = 452).

**Figure 3:**
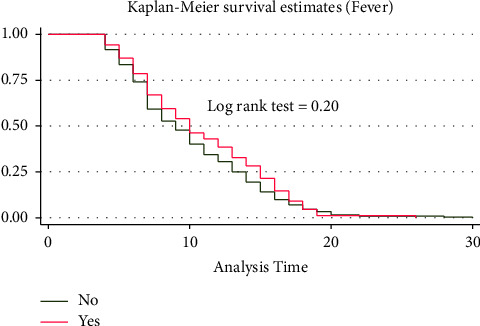
KM survival curve for fever among COVID patients admitted to TGSH, North West Ethiopia, 2022 (*n* = 452).

**Figure 4:**
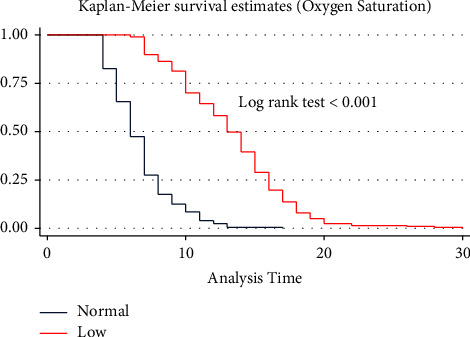
KM survival curve for oxygen saturation among COVID -19 patients admitted to TGSH, North West Ethiopia, 2022 (*n* = 452).

**Figure 5:**
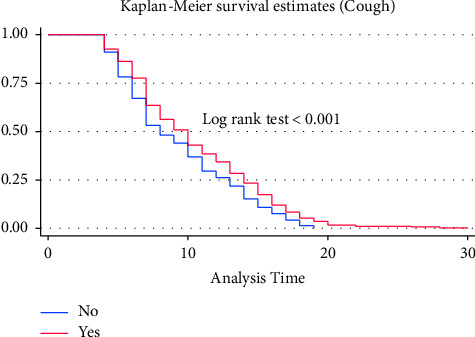
KM survival curve for cough, among COVID patients admitted to TGSH, North West Ethiopia, 2022 (*n* = 452).

A separate Kaplan–Meier graph was drawn to assess the difference in recovery time among categorical predictors. On the Kaplan–Meier survival curve, differences in the median recovery time were observed for predictors like cough, shortness of breath, oxygen saturation, comorbidity, DM, HTN, COVID-19 severity score, hemoglobin level, WBC, PLT level, and use of unfractionated heparin. However, there were no clear differences in the median recovery time for group predictors like sex, residence, blood pressure, fever, headache, chest pain, asthma, and RBC levels. Figures 2–5 demonstrate the Kaplan–Meier graph for some of the predictors.

The log-rank test, on the other hand, was used to assess the whole survival difference between group predictors. Accordingly, predictors such as RR, PR, shortness of breath, oxygen saturation, COVID-19 severity score, history of comorbidity, and hemoglobin and creatinine levels showed differences in the recovery time. During their admission, patients with normal versus low oxygen saturation had the longest median recovery time differences (6 versus 14 days, log-rank test P 0.0001). Males and females, as well as urban and rural residents, had nearly the same median recovery times (10 versus 9 days) (10 versus 9 days). On the other hand, predictors such as chest pain, arthralgia, or fatigue did not affect the median recovery time between the groups (9 days for both groups) **(**see Table 3 attached as a supplementary file).

### 3.5. Model Adequacy Assessment

**Figure 6:**
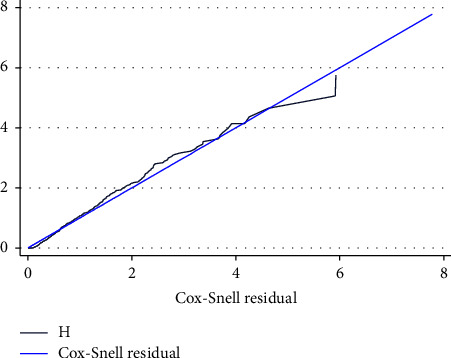
Cox snell residual test for overall adequacy of the model among COVID-19 patients admitted to TGSH, North West Ethiopia, 2022 (*n* = 452).

After fitting the multivariable Cox Proportional Hazard Model, the adequacy of the fitted model was assessed by using Cox Snell residuals. Finally, the graph of the hazard function and the Cox Snell residuals variable was compared to the hazard function to the diagonal line. Figure 6 indicates the cox snell residual of this study. The hazard function follows the 45-degree line, which indicates that the model fitted the data well (Figure 6).

### 3.6. Predictors of Recovery Time from COVID-19

A Cox proportional hazard analysis was fitted to identify significant predictors affecting recovery time. Variables with *p* values less than 0.25 in bivariable Cox proportional analysis and fulfilling the basic requirements of Cox proportional hazard assumptions were included in multivariable Cox proportional hazard analysis. Accordingly, age, pulse rate, respiratory rate, cough, shortness of breath, oxygen saturation, history of preexisting comorbidities, COVID-19 severity score, baseline white blood cell count, hemoglobin level, platelet count, creatinine level, unfractionated heparin (UFH) and corticosteroid use, and oxygen supplementation were candidate variables for the final model. In the multiple variables' Cox proportional hazard analysis, age, oxygen saturation at presentation, shortness of breath, COVID-19 severity score, history of comorbidity, baseline WBC count, and administration of corticosteroids were significant predictors of recovery time.

The age of the patient was one of the significant predictors affecting the median recovery time of COVID-19 patients. A one-year increase in age delayed the recovery time by 2% (AHR = 0.98, 95% CI = 0.97, 0.99). Oxygen saturation at presentation was found to be a significant predictor of recovery time. Low oxygen saturation increased the risk of delayed recovery by 58% compared to patients with normal saturation (AHR = 0.42, CI = 0.31, 0.56). Shortness of breath was one of the respiratory symptoms that affected the recovery time. Patients who presented with shortness of breath had a 35% delayed recovery time compared to those without shortness of breath (AHR = 0.65, 95% CI = 0.50, 0.85). The COVID-19 severity score was also an important factor that affected recovery time. Those patients who came with moderate and severe COVID-19 severity scores had delayed recovery time by 37% (AHR = 0.63, 95% CI = 0.47, 0.85) and 68% (AHR = 0.32, 95% CI = 0.22, 0.47), respectively. Similarly, patients admitted with a history of preexisting comorbidity had a 33% delayed recovery time compared to those patients who had no comorbidity at admission (AHR = 0.67; 95% CI, 0.53, 0.84). Baseline laboratory investigations were also found to be significant predictors of median recovery time among admitted COVID-19 patients. The white blood cell count was one of the baseline investigations affecting recovery time. Patients found to have a high level of white blood cell count had a 35% risk of delayed recovery (AHR = 0.65, 95% CI = 0.49, 0.87). Furthermore, patients who received corticosteroid had a 50% chance of recovering faster than those who did not (AHR = 1.50, 95% CI = 1.19, 1.89) (Table 2).

## 4. Discussion

The purpose of this study was to assess the recovery time and predictors among COVID-19 patients admitted to Tibebe Ghion Specialized Hospital. The median recovery time was 9 days, with a 95 percent confidence interval of 8–10 days. This finding suggested that the median recovery time was longer than in Saudi Arabia (6 days), the University of California San Diego Health (7 days), and an Indian study (7 days**)** [26, 27, 38]. However, the median recovery time was shorter than studies conducted in Italy (19 days), India (25 days), Vietnam (21 days), Iran (13.5 days), China Yun Hospital (13 days), Shenzhen state (21 days), and Wuhan (20 days**)** [18, 20, 21, 39–42]. Similarly, the median recovery time of this study was shorter than previous Ethiopian studies conducted in Millennium (16 days), Yeka Kotebe (19 days), and WURH (17 days) COVID-19 care and treatment centers. [12–14]. The difference might be due to variation in type of facilities and quality of care, period of the pandemic, socioeconomic characteristics, guidelines and policy changes in patient care, changes in admission and discharge criteria, and the differences in early detection of the virus and early treatment. In addition, the differences could be due to a continuous change in the characteristics and pathophysiology of the virus.

Age, oxygen saturation, shortness of breath, COVID-19 severity score at presentation, history of comorbidity, baseline WBC count, and corticosteroid administration were found to be significant predictors of recovery time in a multivariable Cox proportional hazard analysis.

Age was one of the predictors affecting recovery time. This finding was similar to previous studies in Belgium, Vietnam, Korea, Turkey, and China [15–19]. In addition, this study finding was consistent with a study finding in WURH, Ethiopia [14]. The similarities might be due to older age, where people might have suppressed immunity against infections, and the presence of other underlying conditions are more common in older people. Moreover, impaired function and degenerative of the majority of organs, like pulmonary function, is more common in older people. It could also be linked to longer prehospital stays due to older people's poor health-seeking behavior or their reliance on others for medical treatment.

Low oxygen saturation at presentation was found to be significantly associated with recovery time [27]. The finding was consistent with a study conducted in a southern Indian tertiary care hospital. The similarity might be that lower oxygen saturation is related to poor oxygen flow in the tissues and organs as a result of damage to the lung alveoli by the disease. This resulted in impaired function of major organs' ability to respond to the disease. In other words, lower oxygen saturation could be an indication of the disease's severe stage, which could lead to a longer recovery time.

Shortness of breath was one of the respiratory symptoms that also affected the time of recovery. Patients with shortness of breath had a delayed recovery time compared to those without. This finding was consistent with that of studies conducted in Iran and France [20, 22]. This is because patients with SOB might have respiratory-related complications like hypoxia. COVID-19 is a respiratory disease, while shortness of breath is the manifestation of severe respiratory problems. As a result, patients who present with shortness of breath may experience a longer recovery period.

The COVID-19 severity score was an important factor that affected the recovery time. Those patients with moderate and severe COVID-19 severity scores had delayed recovery time compared to patients with mild COVID-19 severity scores. This finding of this study was in line with that of studies conducted in Beijing YouAn hospital and in Karnataka, India. [21, 23]. The similarity might be due to the fact that patients admitted with severe forms of COVID-19 may have reduced pulmonary function, causing the lung and other organs to take longer to recover. Patients with severe COVID-19, on the other hand, may have compromised immunity. Furthermore, patients with severe forms of the disease may have other underlying illnesses.

Another factor that affected the recovery time of COVID-19 patients was the presence of comorbidities. In this study, the presence of a history of comorbidity delayed the recovery time of COVID-19 patients. The finding was consistent with that of studies in South Indian Tertiary Care Hospital, Fancang hospital, China, and Hefei, China [24, 27, 43]. The finding of this study was in line with a study in WURH, Ethiopia, care and treatment center [14]. The similarity might be related to the fact that patients with comorbidities had reduced immunity in common. The presence of comorbidities might also be associated with impaired organ function. However, the finding was in contradicted to a study conducted in Ethiopia's Yeka Kotebe care and treatment center [13]. The differences could be due to the type and severity of comorbidities as well as the medications used. The difference could also be due to the patients' adherence with the treatment prescribed to treat the comorbidity.

Recovery time was also linked to baseline laboratory levels. Patients with a high baseline WBC count in this study had a delayed recovery time. The findings were consistent with a study finding from china [25]. The similarity, which might be an increase in WBC, is an indication of severe form of infection. The infection might be due to COVID-19 itself or other coinfections. When there are co-infections the severity of the disease increases, which might contribute to delayed recovery. Increase WBC on the other hand, might be indication of the worst form of COVID-19.

Corticosteroids were one of the treatments given and affected recovery time. Patients who received corticosteroids recovered more quickly than those who did not. This was in contrast to a multicenter study in China [44]. The differences might be due to the study protocol, the severity of the disease in enrolled patients, or the difference in guidelines for corticosteroid use. The earlier recovery in patients who received corticosteroids in this study might be due to corticosteroids' excellent pharmacological effects on the suppression of exuberant and dysfunctional systemic inflammation. However, there was no conclusive evidence, as some scholars did not recommend the administration to patients with severe viral pneumonia.

### 4.1. Strength and Limitations

We have tried possible models to get the best fitted model to get the best results with precise estimation. The data were extracted from secondary sources, which missed variables related to behavioral factors like smoking, nutritional variables like BMI and others like occupation, which might be potential predictors of recovery time from COVID-19.

## 5. Conclusion

The median recovery time among COVID-19 patients admitted at TGSH COVID-19 care and treatment center was found to be 9 days. The Cox proportional hazard analysis found that older age, low oxygen saturation at admission, shortness of breath, severity of the disease, history of comorbidity, and high level of WBC count were significant predictors of delayed recovery time. On the other hand, corticosteroid use significantly shortens the median recovery time of COVID-19 patients.

Thus, elderly patients and those individuals with low oxygen saturation, shortness of breath, severe COVID-19 illness, and comorbidities have to get closer monitoring and follow-up. It is also important to give due attention to patients with an increased baseline WBC count. Furthermore, the selection of patients for corticosteroid administration has to get special attention.

## Figures and Tables

**Table 1:** Background clinical characteristics (presenting symptoms, comorbidities, and baseline laboratory values) of COVID-19 patients admitted to TGSH, North West Ethiopia, 2022 (*n* = 452).

Variables	Category	*Outcome*		
		Censored	Event	Total
Fever	No	44 (12.6)	305 (87.4)	349 (100)
	Yes	11 (10.7)	92 (89.3)	103 (100)

Cough	No	15 (13.4)	97 (86.6)	112 (100)
	Yes	40 (11.8)	300 (88.2)	340 (100)

SOB	No	25 (10.6)	221 (89.4)	246 (100)
	Yes	30 (14.6)	176 (85.4)	206 (100)

Headache	No	49 (12.8)	334 (87.2)	383 (100)
	Yes	6 (8.7)	63 (91.3)	69 (100)

Chest pain	No	53 (12.8)	362 (87.2)	415 (100)
	Yes	2 (5.4)	35 (94.6)	37 (100)

Myalgia/arthralgia	No	47 (11.80	352 (88.2)	399 (100)
	Yes	8 (15.1)	45 (84.9)	53 (100)

Fatigue	No	50 (12.9)	337 (87.1)	387 (100)
	Yes	5 (7.7)	60 (92.3)	65 (100)

COVID-19 severity score	Mild	8 (9.2)	79 (90.8)	87 (100)
	Moderate	8 (5.3)	142 (94.7)	150 (100)
	Sever	39 (18.1)	176 (81.7)	215 (100)

Comorbidity	No	30 (10.0)	269 (90.0)	299 (100)
	Yes	25 (16.3)	128 (83.7)	153 (100)

WBC	Normal	36 (12)	265 (88.0)	301 (100)
	Low	2 (5.4)	35 (94.6)	37 (100)
	High	17 (14.9)	97 (85.1)	114 (100)

RBC	Normal	39 (11.2)	308 (88.8)	347 (100)
	Low	7 (18.4)	31 (81.6)	38 (100)
	High	9 (13.4)	58 (86.6)	67 (100)

Platelet	Normal	37 (11.3)	291 (88.7)	328 (100)
	Low	18 (15.7)	97 (84.3)	115 (100)
	High	1 (11.1)	8 (88.9)	9 (100)

Hgb	Normal	42 (12.3)	299 (87.7)	341 (100)
	Low	11 (11.7)	83 (88.3)	94 (100)
	High	2 (11.8)	15 (88.2)	17 (100)

Creatinine	Normal	14 (6.8)	192 (93.2)	206 (100)
	Low	6 (13.0)	40 (87.0)	46 (100)
	High	35 (17.5)	165 (82.5)	200 (100)

**Table 2:** Multivariable Cox regression analysis of median recovery time and its predictors among COVID-19 patients admitted in TGSH, North West Ethiopia, 2022 (*n* = 452).

Variables	Category	*Outcome*			CHR 95% CI	AHR (95% CI)	*P*-value
		Censored	Event	Total			
Age		55 (12.2)	397 (87.8)	452 (100)	0.96 (0.95, 0.97)	0.98 (0.97, 0.99)	<0.001

Had normal RR at admission	No	25 (18.7)	109 (81.3)	134 (100)	1	1	0.74
	Yes	30 (9.4)	288 (90.6)	318 (100)	1.98 (1.58, 2.48)	1.05 (0.80, 1.37)	

Had normal PR at admission	No	27 (21.9)	96 (78.1)	123 (100)	1	1	0.39
	Yes	28 (8.5)	301 (91.5)	329 (100)	1.48 (1.17, 1.86)	1.12 (0.87, 1.43)	

Low oxygen saturation	No	17 (7.8)	200 (92.2)	217 (100)	1	1	<0.001
	Yes	38 (16.2)	197 (83.8)	235 (100)	0.16 (0.12, 0.20)	0.42 (0.31, 0.56)	

Cough	No	15 (13.4)	97 (86.6)	112 (100)	1	1	0.27
	Yes	40 (11.8)	300 (88.2)	340 (100)	0.79 (0.62, 0.99)	1.15 (0.89, 1.49)	

SOB	No	25 (10.6)	221 (89.4)	246 (100)	1	1	0.002
	Yes	30 (14.6)	176 (85.4)	206 (100)	0.30 (0.24, 0.36)	0.65 (0.50, 0.85)	

COVID-19 severity score	Mild	8 (9.2)	79 (90.8)	87 (100)	1	1	
	Moderate	8 (5.3)	142 (94.7)	150 (100)	0.55 (0.41, 0.72)	0.63 (0.47, 0.85)	0.002
	Sever	39 (18.1)	176 (81.7)	215 (100)	0.12 (0.09, 0.17)	0.32 (0.22, 0.47)	<0.001

Comorbidity	No	30 (10.0)	269 (90.0)	299 (100)	1	1	
	Yes	25 (16.3)	128 (83.7)	153 (100)	0.51 (0.41, 0.63)	0.67 (0.53, 0.84)	0.001

WBC	Normal	36 (12)	265 (88.0)	301 (100)	1	1	
	Low	2 (5.4)	35 (94.6)	37 (100)	0.69 (0.48, 0.98)	1.23 (0.85, 1.78)	0.27
	High	17 (14.9)	97 (85.1)	114 (100)	0.38 (0.29, 0.48)	0.65 (0.49, 0.87)	0.003

Platelet	Normal	37 (11.3)	291 (88.7)	328 (100)	1	1	
	Low	18 (15.7)	97 (84.3)	115 (100)	0.68 (0.54, 0.85)	0.80 (0.62, 1.03)	0.08
	High	1 (11.1)	8 (88.9)	9 (100)	0.83 (0.43, 1.61)	0.61 (0.29, 1.29)	0.20

Hgb	Normal	42 (12.3)	299 (87.7)	341 (100)	1	1	
	Low	11 (11.7)	83 (88.3)	94 (100)	0.58 (0.45, 0.75)	0.81 (0.62, 1.07)	0.13
	High	2 (11.8)	15 (88.2)	17 (100)	0.85 (0.51, 1.44)	0.87 (0.50, 1.50)	0.61

Creatinine	Normal	14 (6.8)	192 (93.2)	206 (100)	1	1	
	Low	6 (13.0)	40 (87.0)	46 (100)	0.53 (0.37, 0.75)	1.06 (0.72, 1.56)	0.76
	High	35 (17.5)	165 (82.5)	200 (100)	0.55 (0.44, 0.69)	1.04 (0.81, 1.33)	0.78

Oxygen supplemented	No	13 (6.2)	194 (93.7)	207 (100)	1	1	0.64
	Yes	42 (17.1)	203 (82.9)	245 (100)	0.69 (0.56, 0.84)	0.95 (0.75, 1.19)	

Corticosteroid	No	16 (8.8)	165 (91.2)	181 (100)	1	1	0.001
	Yes	39 (14.4)	232 (85.6)	271 (100)	1.54 (1.26, 1.89)	1.50 (1.19, 1.89)	

UFH	No	47 (12.2)	339 (87.8)	386 (100)	1	1	
	Yes	8 (12.2)	58 (87.8)	66 (100)	0.79 (0.49, 0.87)	0.79 (0.57, 1.08)	0.14

## Data Availability

The data were collected from an individual patient medical charts and from the admission and discharge registers. The collected information was only used for the research purpose and was kept confidential. The corresponding author will provide the datasets used and/or analyzed during the current study upon reasonable request.

## Abbreviations

AHR: Adjusted hazard ratio
COVID-19: Corona virus disease 2019
DM: Diabetes mellitus
HGB: Hemoglobin
HTN: Hypertension
PR: Pulse rate
PLT: Platelet
RBC: Red blood cell
SARS: Sever acute respiratory syndrome
TGSH: Tibebe Ghion Specialized Hospital
UFH: Unfractionated heparin
WBC: White blood cells
WHO: World Health Organization.

## Supplementary Materials

Supplementary Materials Table 3. Median Recovery time and Log-rank test among COVID-19 patients admitted to TGSH, North West Ethiopia, 2022 (*n* = 452).
