# Supplementary material for: Time to Recovery from COVID-19 and Its Predictors in Patients Hospitalized at Tibebe Ghion Specialized Hospital Care and Treatment Center, A Retrospective Follow-Up Study, North West Ethiopia
Source: Glob Health Epidemiol Genom. 2023 Sep 12;2023:5586353. doi: 10.1155/2023/5586353 (PMC10508999; doi:10.1155/2023/5586353)
Supplement: Supplementary Materials — Table 3. Median Recovery time and Log-rank test among COVID-19 patients admitted to TGSH, North West Ethiopia, 2022 (n = 452). [file 5586353.f1.docx]

**Supplemented file**

**Table 3. Median Recovery time and Log-rank test among COVID-19 patients admitted to TGSH, North West Ethiopia,2022 (n = 452)**

| Variable | Category | Censored | Event | Total | Median recovery time | Log-rank test (P-value) |
| --- | --- | --- | --- | --- | --- | --- |
| Had normal RR at Admission | No | 25(18.7) | 109(81.3) | 134 (100) | 14 | <0.001 |
|  | Yes | 30(9.4) | 288(90.6) | 318 (100) | 8 |  |
| Had normal PR at admission | No | 27(21.9) | 96(78.1) | 123(100) | 12 | 0.003 |
|  | Yes | 28(8.5) | 301(91.5) | 329(100) | 8 |  |
| Had Normal BP at admission | No | 6(12.2) | 43(87.8) | 49 (100) | 12 | 0.02 |
|  | Yes | 49(12.2) | 354(87.8) | 403(100) | 9 |  |
| Had normal Temperature | No | 13(16.7) | 65(83.3) | 78(100) | 12 | 0.003 |
|  | Yes | 42(11.2) | 332(88.8) | 374(100) | 9 |  |
| Low Oxygen saturation | No | 17(7.8) | 200(92.2) | 217(100) | 6 | <0.01 |
|  | Yes | 38(16.2) | 197(83.8) | 235(100) | 14 |  |
| Fever | No | 44(12.6) | 305(87.4) | 349 100) | 9 | 0.204 |
|  | yes | 11(10.7) | 92(89.3) | 103(100) | 10 |  |
| Cough | No | 15(13.4) | 97(86.6) | 112(100) | 8 | 0.026 |
|  | yes | 40(11.8) | 300(88.2) | 340(100) | 10 |  |
| SOB | No | 25(10.6) | 221(89.4) | 246(100) | 7 | <0.001 |
|  | yes | 30(14.6) | 176(85.4) | 206(100) | 14 |  |
| Headache | No | 49(12.8) | 334(87.2 | 383(100) | 9 | 0.502 |
|  | yes | 6(8.7) | 63(91.3) | 69(100) | 10 |  |
| Chest pain | No | 53(12.8) | 362(87.2) | 415(100) | 9 | 0.786 |
|  | yes | 2(5.4) | 35(94.6) | 37(100) | 9 |  |
| Arthralgia | No | 47(11.8) | 352(88.2) | 399(100) | 9 | 0.538 |
|  | yes | 8(15.1) | 45(84.9) | 53(100) | 9 |  |
| Fatigue | No | 50(12.9) | 337(87.1) | 387(100) | 9 | 0.779 |
|  | yes | 5(7.7) | 60(92.3) | 65(100) | 9 |  |
| Covid-19 severity score | Mild | 8(9.2) | 79(90.8) | 87 (100) | 6 | < 0.001 |
|  | Moderate | 8(5.3) | 142(94.7) | 150(100) | 7 |  |
|  | Sever | 39(18.1) | 176(81.7) | 215(100) | 14 |  |
| Comorbidity | No | 30(10.0) | 269(90.0) | 299(100) | 7 | < 0.001 |
|  | yes | 25(16.3) | 128(83.7) | 153(100) | 13 |  |
| Hypertension | No | 45(11.5) | 345(88.5) | 390(100) | 8 | < 0.001 |
|  | yes | 10(16.1) | 52(83.9) | 62(100) | 14 |  |
| Diabetes Mellitus | No | 41(10.8) | 339(89.2) | 380 (100) | 8 | < 0.001 |
|  | Yes | 14(19.4) | 58(80.6) | 72(100) | 12 |  |
| Asthma | No | 52(12.1) | 378(87.9) | 430(100) | 9 | 0. 019 |
|  | Yes | 3(13.6) | 19(85.4) | 22(100) | 14 |  |
| Chronic Heart  Disease | No | 53(12.2) | 380(87.8) | 433(100) | 9 | 0.025 |
|  | Yes | 2(10.5) | 17(89.5) | 19(100) | 14 |  |
| HIV | No | 52(11.8) | 390(88.2) | 442(100) | 9 | 0.109 |
|  | Yes | 3(30) | 7(70) | 10(100) | 14 |  |
| WBC | Normal | 36 (12) | 265 (88.0) | 301 (100) | 7 | < 0.001 |
|  | Low | 2 (5.4) | 35 (94.6) | 37 (100) | 10 |  |
|  | High | 17 (14.9) | 97 (85.1) | 114 (100) | 14 |  |
| RBC | Normal | 39 (11.2) | 308 (88.8) | 347 (100) | 8 | 0..002 |
|  | Low | 7 (18.4) | 31 (81.6) | 38 (100) | 10 |  |
|  | High | 9 (13.4) | 58 (86.6) | 67 (100) | 13 |  |
| HGB | Normal | 42 (12.3) | 299 (87.7) | 341 (100) | 8 | < 0.001 |
|  | Low | 11 (11.7) | 83 (88.3) | 94 (100) | 12 |  |
|  | High | 2 (11.8) | 15 (88.2) | 17 (100) | 10 |  |
| PLT | Normal | 37 (11.3) | 291 (88.7) | 328 (100) | 8 | <0.001 |
|  | Low | 18 (15.7) | 97 (84.3) | 115 (100) | 12 |  |
|  | High | 1 (11.1) | 8 (89.9) | 9 (100) | 12 |  |
| Creatinine | Normal | 14 (6.8) | 192 (93.2) | 206 (100) | 7 | < 0.001 |
|  | Low | 6 (13.0) | 40 (87.0) | 46 (100) | 11 |  |
|  | High | 35 (17.5) | 165 (82.5) | 200 (100) | 12 |  |
| Antibiotics | No | 7(6.1) | 107(93.9) | 114 (100) | **12** | 0.007 |
|  | Yes | 48(14.2) | 290(85.8) | 338 (100) | **8** |  |
| Corticosteroid | No | 16(8.8) | 165(91.2) | 181(100) | 12 | <0.001 |
|  | Yes | 39(14.4) | 232(85.6) | 271(100) | 8 |  |
| UFH | No | 47(12.2) | 339(87.8) | 386 (100) | 9 | 0.01 |
|  | Yes | 8(12.2) | 58(87.8) | 66 (100) | 12 |  |
| Oxygen supplemented | No | 13(6.2) | 194(93.7) | 207(100) | 7 | < 0.001 |
|  | Yes | 42(17.1) | 203(82.9) | 245(100) | 10 |  |
